# Morphology and function of cryopreserved whole ovine ovaries after heterotopic autotransplantation

**DOI:** 10.1186/1477-7827-6-16

**Published:** 2008-04-11

**Authors:** Anna T Grazul-Bilska, Jashoman Banerjee, Ilker Yazici, Ewa Borowczyk, Jerzy J Bilski, Rakesh K Sharma, Maria Siemionov, Tommaso Falcone

**Affiliations:** 1 Department of Animal Sciences, North Dakota State University, Fargo, ND, USA; 2 Obstetrics, Gynecology and Women's Health Institute, Cleveland Clinic, Cleveland, OH, USA; 3 Department of Plastic Surgery, Cleveland Clinic, Cleveland, OH, USA

## Abstract

**Background:**

The objective of this study was to perform complex characterization of cryopreserved and then autotransplanted ovaries including determination of the ability to respond to in vivo follicle stimulating hormone (FSH)-treatment, fertilizability of retrieved oocytes, and morphology, vascularization, cellular proliferation and apoptosis in sheep.

**Methods:**

Mature crossbred ewes were divided into two groups; an intact (control) group (n = 4), and autotransplanted group (n = 4) in which oophorectomy was performed laparoscopically and ovaries with intact vascular pedicles frozen, thawed and transplanted back into the same animal at a different site. Approximately five months after autotransplantation, estrus was synchronized, ewes were treated with FSH, and ovaries were collected. For all ovaries, number of visible follicles was determined, and collected cumulus oocyte complexes (COC) were matured and fertilized in vitro. Remaining ovarian tissues were fixed for evaluation of morphology, expression of factor VIII (marker of endothelial cells), vascular endothelial growth factor (VEGF; expressed by pericytes and smooth muscle cells), and smooth muscle cell actin (SMCA; marker of pericytes and smooth muscle cells), and cellular proliferation and apoptosis. Two fully functional ovaries were collected from each control ewe (total 8 ovaries).

**Results:**

Out of eight autotransplanted ovaries, a total of two ovaries with developing follicles were found. Control ewes had 10.6 +/- 2.7 follicles/ovary, oocytes were in vitro fertilized and developed to the blastocyst stage. One autotransplanted ewe had 4 visible follicles from which 3 COC were collected, but none of them was fertilized. The morphology of autotransplanted and control ovaries was similar. In control and autotransplanted ovaries, primordial, primary, secondary, antral and preovulatory follicles were found along with fully functional vascularization which was manifested by expression of factor VIII, VEGF and SMCA. Proliferating cells were detected in follicles, and the rate of apoptosis was minimal in ovaries of control and autotransplanted ovaries.

**Conclusion:**

These data demonstrate successful autotransplantation of a portion of frozen/thawed ovaries manifested by restoration of selected ovarian function including in vitro maturation of collected oocytes, presence of follicles from several stages of folliculogenesis and blood vessels expressing specific markers of vascularization, and proliferation and apoptosis of ovarian cells. Thus, heterotopic autotransplantation of a whole frozen/thawed ovary allows for development of preovulatory follicles, oocyte growth, and for restoration of vascularization and cellular function. However, additional improvements are required to enhance the efficiency of autotransplantation of frozen/thawed ovaries to produce more oocytes.

## Background

Therapeutic advances in childhood and adult cancers are leading to improved survival and cures [1]. The exposure of ovaries and uterus to radiotherapy and chemotherapy in childhood or the reproductive years predispose them to premature ovarian failure and permanent damage so that survivors of cancer are devastated by the physical, psychological, and social consequences of functional castration [2-4]. Therefore, to preserve fertility numerous attempts have been made to optimize procedures of human ovarian tissues preservation including cryopreservation of oocytes, cortical tissues and whole ovaries followed by orthotopic or heterotopic grafting [5-18]. However, using human tissues for optimization and improvement of specific procedure is frequently impractical or unethical [19]. Therefore, several animal models including the sheep model have been developed to study ovarian cryopreservation and grafting [20-27].

It has been reported by us and others that autotransplantation of whole fresh or cryopreserved sheep ovaries with vascular anastomosis is technically feasible [5,20,23,24,26,28-30]. In addition, we have demonstrated that autotransplantation of fresh ovaries to the deep inferior epigastric vessels provides good short-term results [23,24]. However, long term survival and function of these ovaries has not been previously investigated. Furthermore, characterization of whole frozen/thawed and autotransplanted ovaries have not been performed in detail.

We hypothesized that the fertilizing potential of oocytes and vascularization of the whole ovary with microvascular anastomosis after freezing/thawing and heterotopic autotransplantation can be restored. Therefore, the objectives of this complex study were to evaluate (1) the ability of ovaries to respond to in vivo FSH-treatment, (2) fertilizability of oocytes retrieved from cryopreserved autotransplanted ovaries, and (3) morphology, vascularization, cellular proliferation and apoptosis in cryopreserved and heterotopically autotransplanted ovaries.

## Methods

### Animals

This study was approved by Institutional Animal Research Committee of the Cleveland Clinic, and by the Institutional Animal Care and Use Committee of North Dakota State University (NDSU). Eight adult, nonpregnant Dorset/Suffolk crossbred ewes, weighing 50–60 kg, 2–4 years old were used. Animals were divided into two groups: an intact (control) group (n = 4), and autotransplanted group (n = 4) in which oophorectomy was performed laparoscopically and ovaries with intact vascular pedicles frozen, thawed and transplanted back into the same animal at a different site. For one ewe serving as an additional control, immediately after oophorectomy, not cryopreserved ovaries were autotransplanted at a different site. For all ewes, surgical procedures and cryopreservation were performed during seasonal anestrus in May-June, but hormonal stimulation and collection of ovaries during reproductive season in October.

### Surgical technique

After induction of general anesthesia with halothane (2.0 vol. % in oxygen) and mechanical ventilation (endotracheal intubation), the ewe was secured on the operating table. A 10-mm 0 degree laparoscope was inserted through an incision half an inch below the umbilicus. Pneumoperitoneum was created with CO_2 _at 10 liter/min and 12 mm Hg of intraperitoneal pressure. The animal was placed in a slight Trendelenburg position, and the pelvic cavity was inspected. Ancillary 5-mm trocars were inserted through the right and left lateral skin incisions 5 cm below and 8 cm lateral to the umbilicus. A third 5-mm trocar was placed at the level of the camera port. The ovarian vessels were identified, and their course was traced from the hilum cephalad. The ovary was dissected off the surrounding tissue. The skeletonized blood vessels were double ligated as proximal as possible with non-absorbable 1-0 multifilament silk suture using intracorporeal ligature technique. The ovary was removed through one of the 5-mm trocars and cannulated [23]. Two ovaries from each sheep were removed and then prepared for freezing.

### Cryopreservation

The ovarian vessels and excess hilar tissue were dissected, and ovarian ligaments were trimmed after oophorectomy. The grafts were perfused via the ovarian artery with heparinized (5 IU/ml; w/v) Ringer's solution for 10 minutes, followed by perfusion of cryoprotectant containing Leibovitz L-15 medium (Irvine Scientific, Santa Ana, CA), 10% fetal bovine serum (FBS; v/v; Irvine Scientific), and 1.5 M dimethyl sulfoxide (DMSO; v/v; Sigma, St. Louis, MO) for 5 minutes using Horizon Nxt. Modular Infusion system (McGaw Inc., Irvine, CA) to maintain a flow rate at 1.3 mL/min with continuous replenishment of the reservoir. After perfusion, the ovary was transferred to a 5 mL 12.7 × 92 mm cryovial (Corning Coaster Corporation, Cambridge, MA) containing the cryoprotective mixture for controlled freezing using Planer Cryochamber (Planer Freezer Ltd., Middlesex, United Kingdom). Cooling started at 4°C and continued at 2°C/min until ice nucleation was induced at -7°C (seeding). The temperature was then reduced at 0.3°C/min until -40°C and, subsequently, at 25°C/min until -140°C before the cryovials were plunged into liquid nitrogen [23,24].

### Thawing

After 1 week, the vial was removed from the liquid nitrogen tank and held for 1 minute at room temperature before plunging and swirling it in a water bath at 37°C with gentle shaking. The contents of the vial were quickly emptied into a petri dish containing Leibovitz L-15 medium supplemented with 10% FBS. The ovary was washed in medium and immediately perfused with Leibovitz L-15 medium supplemented with 10% FBS and several steps of sucrose (0.25 M, 0.1 M, and 0 M) at 3 mL/min for 30 minutes. Perfusion times were 10 minutes for each step [23,24].

### Autotransplantation

An abdominal incision (3–4 cm) of the animal that had undergone oophorectomy was made on the anterior abdominal wall, near the site of the 5 mm trocar, in order to expose the deep inferior epigastric vessels. A branch with a diameter matching that of the ovarian vessels was identified and dissected. Standard microsurgical instrumentation and Acland vascular clamps were used for anastomosis. Each anastomosis was 8 to 10 interrupted sutures with 9-0 or 10-0 Prolene (US Surgical Co., Norwalk, CT) tied in an end-to-end fashion under a Zeiss surgical microscope (Carl Zeiss, Oberikochen, Germany). Diluted heparin and papaverine were used topically as required. The ovary was attached between bundles of the rectus muscle. After removal of the clamps, the site of anastomosis was inspected for blood flow (Patency) for at least 20 minutes. The same process was repeated on other site of the abdomen for the opposite ovary. After surgery, all animals received s.c. 5,000 IU of sodium heparin (Eli Lilly and Company, Indianapolis, IN) for 3 days [23,24].

### Post operative care, hormonal treatment, and blood samples and tissue collection

All sheep were housed in the animal facility setting for 2 weeks after transplantation in Ohio. The surgical sites were examined twice daily for each autotransplanted ewe. Then all sheep were transported to the Animal Nutrition and Physiology Center at NDSU, and kept in individual pens.

Blood samples were collected at the time of autotransplantation (collection 1), then every 4 weeks (collections 2 and 3), at the time of Chronogest (progestagen) sponge (30 mg flugestone acetate/sponge; Intervet, UK) insertion (collection 4) and removal 14 days later (collection 5), and on days 15, 16 and 17 after sponge removal, which corresponded with days 13, 14 and 15 of the estrous cycle, respectively, in control ewes. Serum samples were used for determination of FSH, LH, and estradiol-17β (E2) in peripheral blood by using Immulite technology (Immulite 1000; Diagnostic Product Corporation, Los Angeles, CA). All ewes were checked for behavioral estrus twice daily using a vasectomized ram for 72 h after Chronogest sponge withdrawal [31]. Ewes received twice daily (morning and evening) injections of FSH-P (Sioux Biochemical, Sioux Center, IA, USA) on day 15 (5 units/injection) and 16 (4 units/injection) after Chronogest sponge withdrawal, as described previously [31,32].

On day 17 after Chronogest sponge withdrawal, which corresponded to day 15 of the estrus cycle in control ewes, ovaries were collected from all ewes. Number of follicles visible on the ovarian surface was determined and cumulus oocyte complexes (COC) were collected. Oocytes were then matured and fertilized in vitro, as described below. The remaining ovarian tissues were cut into half, then one half was fixed in 10% formalin solution and another half in Carnoy's solution for histological, immunohistochemical and/or is situ hybridization (TUNEL) evaluations.

### In vitro maturation, fertilization and culture

Oocytes were matured and fertilized in vitro as described in detail before [31-33]. Briefly, COC were incubated (39°C, atmosphere 5% CO_2 _and 95% air) in maturation medium for 24 h, transferred to fertilization medium, and incubated (39°C, 5% O_2_, 5% CO_2 _and 90% N_2_) with fertility proven sperm (0.5–1 million sperm/ml; [31-34]). After 22 h, presumptive zygotes were transferred to culture medium without glucose, and cleavage rates were determined 48 h later. Embryos were then transferred to the fresh culture medium with glucose. The rate of cleavage (number of cleaved vs. non-cleaved oocytes), and the rate of early embryonic development (time and percentage reaching the stage of morula or blastocyst) were evaluated every second day during an 8 day culture. Unfertilized oocytes were fixed in methanol followed by DAPI staining and evaluation of maturation status under epifluorescent microscopy [35,36].

### Histochemistry, immunohistochemistry and in situ hybridization in ovarian tissues

To assess ovarian morphology, tissue slides were stained with hematoxyline and periodic acid Schiff's as previously described [37].

Vascularization was determined by immunolocalization of factor VIII (marker of endothelial cells), VEGF (expressed by pericytes and smooth muscle cells), and SMCA (marker of pericytes and smooth muscle cells). Detection of factor VIII, VEGF, SMCA and proliferating cell nuclear antigen (PCNA) was performed as previously described [38-41]. Briefly, sections of ovarian tissues fixed in Carnoy's solution were rinsed several times in PBS containing Triton-X100 (0.3%, v/v) and then were treated for 20 min with blocking buffer [PBS containing normal goat serum (1–2%, v/v)] followed by treatment with primary antibody against factor VIII (rabbit polyclonal; Sigma, St. Louis, MO), VEGF (a peptide affinity-purified anti-rabbit serum raised against VEGF peptide; [39]), SMCA (mouse monoclonal; Oncogene Research Products; San Diego, CA) or PCNA (mouse monoclonal; Zymed, San Francisco, CA) overnight at 4°C. Primary antibody was detected by using a biotinylated secondary antibody and a streptavidin-biotin-peroxidase complex (Vector Laboratories, Burlingame, CA). All sections were counterstained with fast red (Sigma) to visualize cell nuclei. Control sections were incubated with normal mouse or rabbit serum in place of primary antibody. To localize apoptotic cells in ovarian tissue sections fixed in formalin, TdT-FragEL™, DNA fragmentation detection kit was used according to the manufacturer's protocol (Oncogene Research Products, San Diego, CA; [40]).

## Results

**Figure 1:**
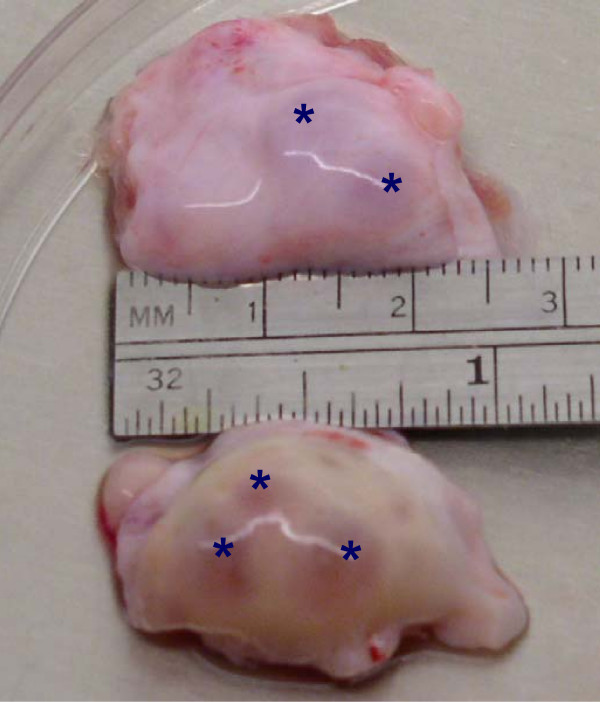
Representative micrograph of ovaries collected from autotransplanted (top ovary) and control (bottom ovary) ewes. Stars (*) indicate large follicles.

**Figure 2:**
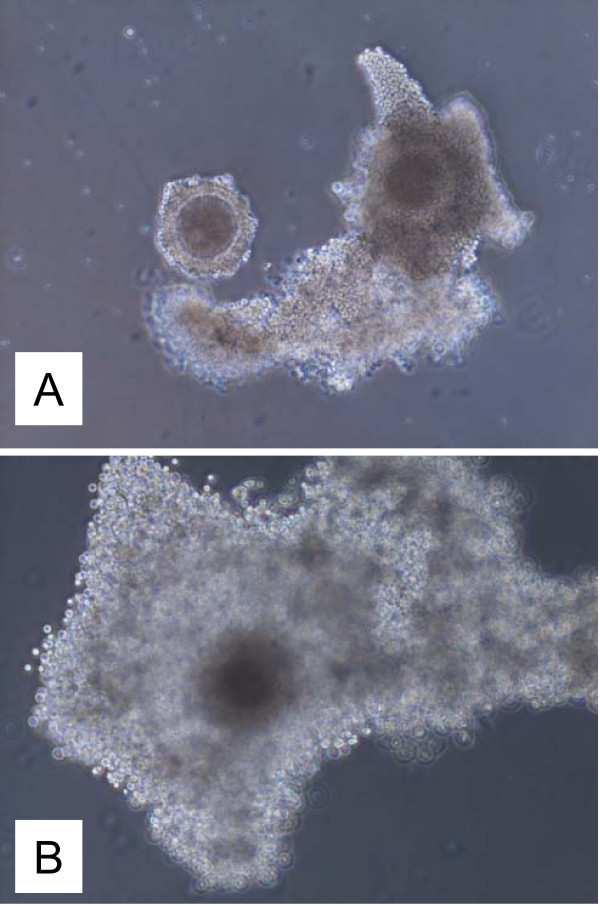
Image of oocytes collected from an autotransplanted ewe before (A) and after (B) maturation. Magnification 100×.

After removal of Chronogest sponges, all control but none of the autotransplanted ewes expressed behavioral estrus. Out of 8 autotransplanted cryopreserved ovaries (n = 4 ewes) two functional ovaries (25%) were collected (one ovary in each of two ewes; 50%). The ovaries were consider functional when (1) a size of ovary was similar to the size of control ovaries, (2) visible follicle(s) were present on ovarian surface, and/or (3) invisible follicles were present in the ovary as determined using histology. The size of the ovaries from autotransplanted and control ewes was similar (Fig. 1). FSH-treatment induced development of follicles in two autotransplanted and all control ewes. One autotransplanted ewe had 2 large (5 mm) and 2 small (2 mm) follicles visible on the ovarian surface (Fig. 1), but another autotransplanted ewe did not have follicles visible on ovarian surface. However, follicles in this ovary were detected in histological sections. For control ewes, the mean number of large (>3 mm) and small (≤ 3 mm) follicles/ovary was 6.4 ± 1.4 and 4.2 ± 1.7 (means ± SEM) respectively, and fertilization and blastocyst formation rates were 89 ± 3% and 39 ± 4%, respectively. Three COC from one autotransplanted ewe were collected. Morphologically, two oocytes looked healthy before and after maturation (Fig. 2), but one oocyte looked atretic (not shown). Healthy-looking oocytes had several layers of cumulus cells, but atretic-looking oocytes had a very few cumulus cells attached, cytoplasm was not uniform, and perivitelline space was enlarged. Two healthy-looking oocytes matured in vitro by reaching metaphase II stage (as detected by DAPI staining; data not shown because images of oocytes before and after maturation have been published by us before; [35,36]), but none of them was fertilized. For one ewe autotransplanted with not cryopreserved (fresh) two ovaries, one ovary was collected (50%), with a size similar to presented on Fig. 1 with two visible follicles 4 and 5 mm in diameter. One healthy-looking oocyte was collected from this ovary which matured in vitro but was not fertilized in vitro.

**Figure 3:**
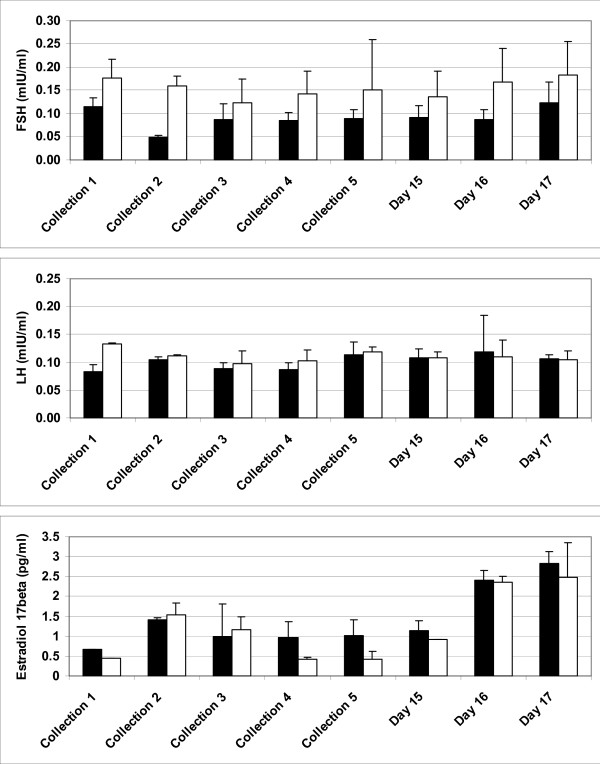
**Concentrations (mean ± SEM) of FSH (mIU/ml), LH (mIU/ml) and E2 (pg/ml) in serum of control (n = 4; black bars), and autotransplanted (n = 2; open bars) ewes with partially restored ovarian function before FSH-treatment, and on days 15, 16 and 17 after removal of Chronogest sponges.** Collection 1 was at the time of autotransplantation, collections 2 and 3 were approximately every 4 weeks within the first two months after autotransplantation, collection 4 was at Chronogest sponge insertion, collection 5 was at sponge withdrawal, and remaining collections were on days 15, 16 and 17 after sponge withdrawal, which corresponds to days 13–15 of the estrous cycle in control ewes.

Concentrations of FSH, LH and E2 in peripheral blood of controls and two autotransplanted ewes with functional ovaries is shown on Fig. 3. We did not perform statistical analysis since only 2 autotransplanted ewes had functional ovaries. Peripheral FSH concentration seems to be greater in autotransplanted ewes that control ewes, but LH concentration seems to be similar in both groups. Estradiol-17β concentration seems to be enhanced in autotransplanted and control ewes during FSH-stimulation (Fig. 3; Days 15–17). Concentration of hormones in peripheral blood of ewe with autotransplanted not cryopreserved ovaries (data not shown) followed a pattern for other autotransplanted ewes demonstrated on Fig. 3.

**Figure 4:**
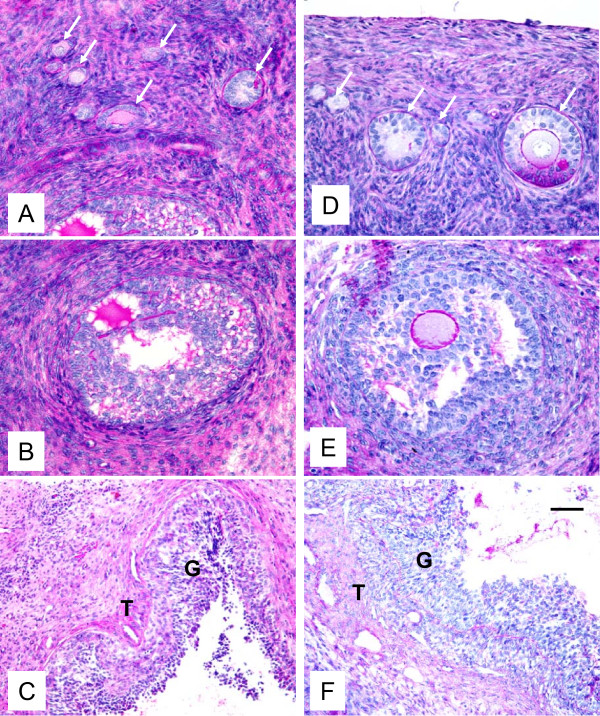
**Representative micrograph of ovarian sections from autotransplanted (A, B, C; left column) and control (D, E, F; right column) ewes stained with hematoxylin and periodic acid Schiff's reagent.** Note presence of primordial, primary and secondary follicles (arrows, A and D), early antral follicles (B and E), and preovulatory follicle (C and F). G = granulosa and T = theca in preovulatory follicles in C and F. Bar = 50 μm for A, B, D, E and 100 μm for C and F.

Histological evaluation demonstrated that the morphology of control and autotransplanted ovaries was similar. In autotransplanted and control ovaries, primordial, primary, secondary, antral and preovulatory follicles were found (Fig. 4). In one autotransplanted ovary, the CL was present which indicates that one follicle ovulated, likely after Chronogest sponge removal.

**Figure 5:**
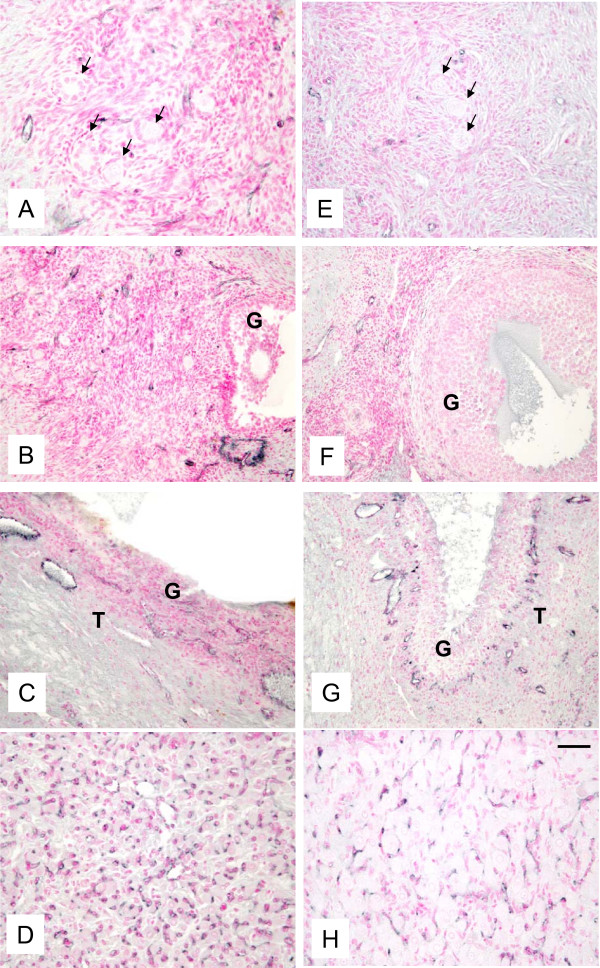
**Representative micrograph of staining for factor VIII (black color) in ovarian tissues from autotransplanted (A, B, C, D; left column) and control (E, F, G, H; right column) ewes.** Note the presence of factor VIII in blood vessels in the area containing primordial/primary (A, C) and antral follicles (B, F), in the theca layer of preovulatory follicles (C, G), and in capillaries of the CL (D, H). Arrows indicate primordial/primary follicles. G = granulosa and T = theca in preovulatory follicles in B, C, F and G. Control sections did not exhibit any positive staining (see Fig. 8 insert). Bar = 50 μm for A, C, D, E, G, H and 100 μm for B and F.

**Figure 6:**
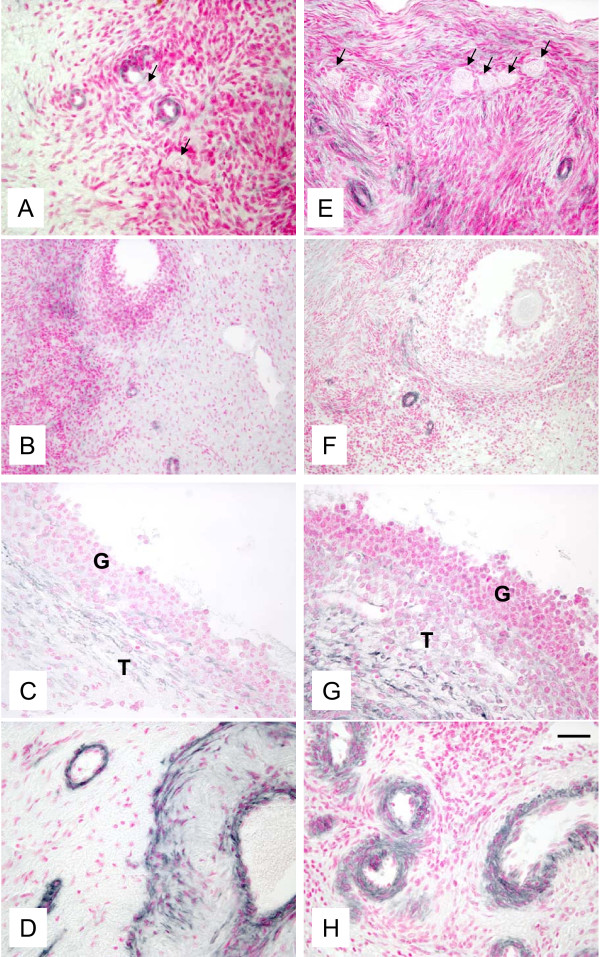
**Representative micrograph of staining for VEGF (black color) in ovarian tissues from autotransplanted (A, B, C, D; left column) and control (E, F, G, H; right column) ewes.** Note presence of VEGF in blood vessels in the area containing primordial/primary and antral follicles (A, B, E, F), in the theca layer of preovulatory follicles (C, G), and in the large blood vessels in the ovarian stroma (D, H). Arrows indicate primordial/primary follicles. Control sections did not exhibit any positive staining (see Fig. 8 insert). Bar = 50 μm for A, B, D, E, G, H and 100 μm for B and F.

**Figure 7:**
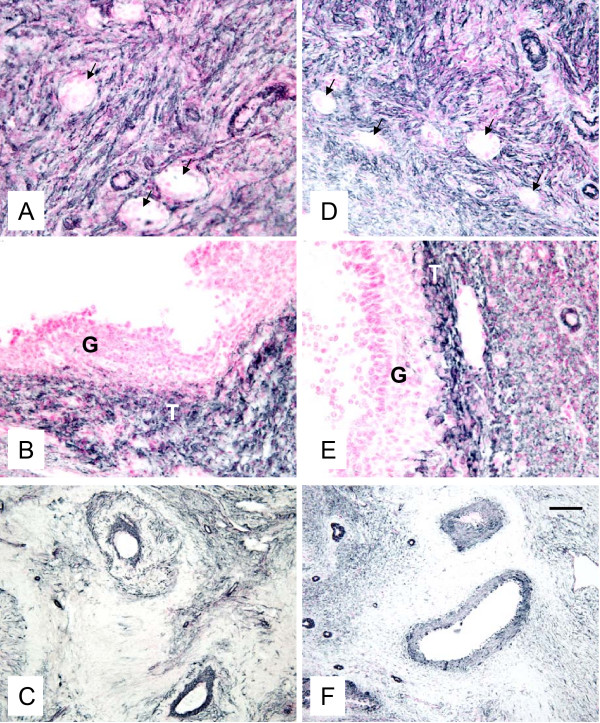
**Representative micrograph of staining for SMCA (black color) in ovarian tissues from autotransplanted (A, B, C; left column) and control (D, E, F; right column) ewes. **Note presence of SMCA in blood vessels and connective tissue in the area containing primordial/primary/secondary follicles (A, D), in the thecal layer of preovulatory follicles (B, E), and in larger and smaller blood vessels of ovarian stroma (C, F).  Arrows indicate primordial/primary/secondary follicles. G = granulosa in preovulatory follicles in B and E. Control sections did not exhibit any positive staining (see Fig. 8 insert). Bar = 50 µm for A, B, D, G and 100 µm for C and F.

Presence of fully functional vascularization was manifested by expression of factor VIII (Fig. 5), VEGF (Fig. 6) and SMCA (Fig. 7) in both autotransplanted and control ovaries. The pattern of staining for factor VIII, VEGF and SMCA was similar for autotransplanted and control ovaries (Fig. 5, 6, 7). Factor VIII was detected in blood vessels in areas containing primordial/primary (Fig. 5A,E), small antral (Fig. 5B,F) and preovulatory (Fig. 5C,G) follicles, and in the corpora lutea (Fig. 5D,H) in autotransplanted and control ovaries. VEGF was also detected in ovarian blood vessels in the area containing primordial/primary (Fig. 6A,E), small antral (Fig. 6B,F) and preovulatory follicles (Fig. 6C,G), and in the larger blood vessels of ovarian medulla, cortex and hilus (Fig. 6D,H) in autotransplanted and control ovaries. SMCA was detected in blood vessels and connective tissue/extracellular matrix in the area containing primordial/primary/secondary (Fig. 7A,D), small antral (not shown) and preovulatory (Fig. 7B,E) follicles, and in the larger blood vessels of ovarian medulla, cortex and hilus (Fig. 7C,F) in autotransplanted and control ovaries.

**Figure 8:**
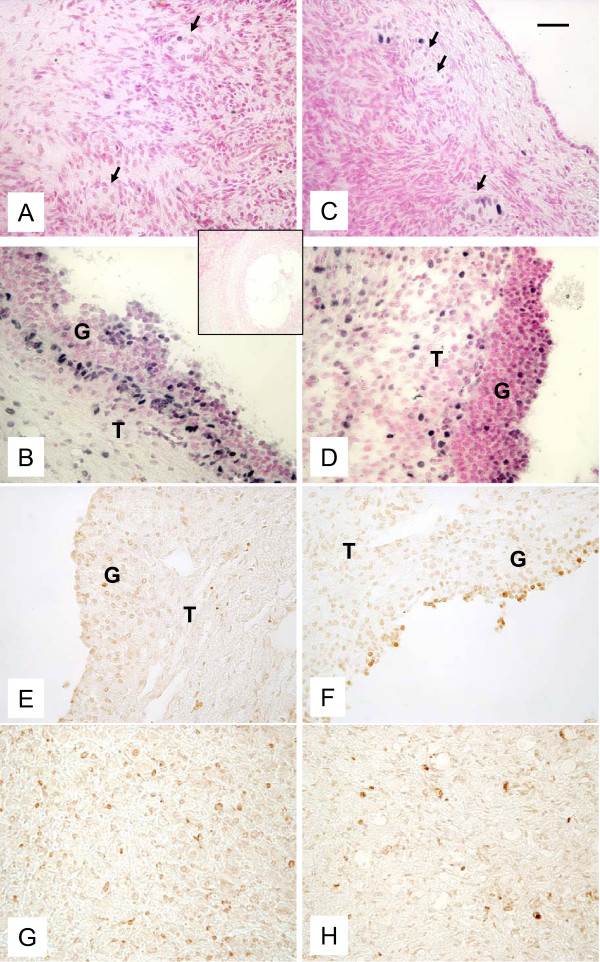
**Representative micrograph of staining for PCNA (black color; A-D) and apoptotic cells (brownish color; E-H) in ovarian tissues from autotransplanted (A, B, E, G; left column) and control (C, D, F, H; right column) ewes.** Note the presence of PCNA in primordial/primary (arrows; A, C) and preovulatory follicles (B, D), and apoptotic cells in granulosa layer of preovulatory follicle (E, F) an in the CL (G, H). G = granulosa and T = theca in preovulatory follicles in B, D, E and F. Control sections did not exhibit any positive staining (insert). Bar = 50 μm.

Proliferating cells were detected in primary and secondary (Fig. 8A,C), small antral (data not shown) and preovulatory follicles (Fig. 8B,D) in control and autotransplanted ovaries. The apoptotic cells/bodies were not detected in the areas containing primordial/primary, secondary and small antral follicles (data not shown), but apoptosis was detected in some granulosa cells of preovulatory follicles (Fig. 8E,G) and in the CL (Fig. 8F,H) in autotransplanted and control ovaries. Presence of some apoptotic cells is typical for the preovulatory follicles [42] and for the CL undergoing functional luteolysis [40], which likely was occurring on day 15 of the estrous cycle in control and possibly autotransplanted ewes. The pattern of staining for PCNA and apoptotic cells was similar in autotransplanted and control ovaries (Fig. 8).

For one ewe autotransplanted with not cryopreserved ovaries, the morphology of ovarian sections, and staining for the presence of markers of vascularization, and cell proliferation and apoptosis was similar to those observed for other autotransplanted ewes (data for this ewe are not shown, but are similar to those presented on Fig. 4, 5, 6, 7, 8).

## Discussion

Although tremendous progress has been made in a recent years in human ovarian tissue cryopreservation and autotransplantation [5,10,12,13,17], there is a constant need for improvement of these techniques. Therefore, several animal models including sheep, rodents, rabbits and monkeys have been developed to optimize both cryopreservation and transplantation of preserved ovarian tissues or whole ovaries [12,17,20-22,43].

The present study demonstrated that ovarian function was partially restored after heterotopic autotransplantation of frozen/thawed whole ovine ovaries. Other studies using animal models also demonstrated that whole ovarian freezing may be an acceptable method for ovarian preservation, and subsequent orthotopic or heterotopic transplantation [24,26,29,44]. In fact, orthotopic transplantation of frozen or vitrified/thawed ovine ovaries or hemi-ovaries resulted in the successful development of oocytes which were then developed either to early parthenogenetic embryos [30] or to live births [26,45,46].

In animal model, whole ovary has been transplanted orthotopically [26,30] and heterotopically [23]. The orthotopic transplantation requires major surgery to access the ovarian vessels, especially since the diameter of ovarian artery in several animal models and human is about 1 mm. In addition, orthotopic transplantation caused massive loss of follicles (more than 90%), but resulted in successful pregnancy [26]. Furthermore, after orthotopic autotransplantation, reanastomosis to the ovarian vessels failed in 3 of 8 sheep (38%), severe adhesions in one and mild in three of the five animals were revealed through laparotomy two months after transplantation, and normal progesterone cyclicity was seen in only 37% of sheep [29]. These researchers have also stated that the method of end-to-end anastomosis into either the original site or to the pedicle of the contralateral ovary "is far more challenging technically than transplantation to superficial vessels in the abdominal wall" [30]. Due to these difficulties, the method of transplantation to the neck rather than to the original site was recommended [29]. But we proposed and have implemented transplantation to even easier accessible site – the anterior abdominal wall with easy access to the inferior epigastric vessels [preset study, [23,24]]. Although both types of transplantations may result in restoration of some ovarian function, heterotopic transplantation seems to be easier to perform due to easier access to site for transplantation which may result in faster recovery. Thus, we postulate that heterotopic autotransplantation to the site on the abdominal wall would allow for easy and successful creation of vascular anastomosis of transplanted ovaries due to less invasive surgery, and a recovery after such surgery would be likely faster than after orthotopic transplantation. In human clinical cases, ovarian tissues but not whole ovaries have been transplanted both orthotopically [10-12] and heterotopically [47] resulting in occasional live birth.

In the current study, approximately 5 months after autotransplantation, the size of ovaries at the time of oocyte collection was similar to control ovaries, and preovulatory follicles were visible on the ovarian surface. In another study, ovaries were normal in appearance and had visible follicles and vascularization 18–19 months after orthotopic transplantation in sheep [26]. Furthermore, pregnancy outcome seemed to be greater when levels of progesterone increase was observed more that 40 weeks than 17–26 weeks after autotransplantation of vitrified hemi-ovaries in sheep [48]. Meirow et al. [49] demonstrated that nine months after transplantation of ovarian tissues but not in earlier months (e.g., 2, 3 or 8), a mature oocyte was recovered in human patient. These and other [22,26,50] data indicate that rather longer period of time (several months) but not short time is required to restore ovarian function after freezing/thawing and transplantation.

In our study, oocytes collected from visible follicles from autotransplanted ovaries had normal morphology, progressed to metaphase II stage after in vitro maturation, but in vitro fertilization (IVF) did not occur. This indicates that after whole ovary freezing/thawing and autotransplantation, follicles are able to respond to FSH treatment and grow to preovulatory stage, and healthy looking oocytes which matured in vitro can be produced. Lack of IVF in our experiment could be due to several factors including freezing/thawing procedures, transplantation surgical technique and oocyte culture conditions. Several protocols used for freezing/thawing of whole sheep ovaries have been successfully tested by us and others [24-27,30,44,51], but these protocols still have some limitations which include selection of freezing and thawing medium, ovarian perfusion, and size of cryovials. Although we have optimized freezing/thawing and autotransplantation procedure and used it in several experiments [17,23,24], not all cryopreserved autotransplanted ovaries regained their function, and a portion of them became atretic. This indicates that additional improvements in these protocols should be made to enhance ovarian survival. It appeared that, in the present experiment, the size of cryovials was not creating an additional problem, since freezing/thawing and autotransplantation were performed during seasonal anestrus, when size of ovine ovaries in small. However, larger cryovials are desired to cryopreserve human ovaries or sheep ovaries from reproductive season. Furthermore, larger ovaries possess larger blood vessels which facilitate suturing during transplantation surgery [17]. Finally, the culture conditions of in vitro maturation and fertilization could contribute to lack of fertilization in our experiment. In sheep, the fertilization rate in vitro is within range of 60–80% but not 100% [32-34]. Thus, some oocytes are not fertilized even when oocytes are collected from fresh ovaries. In this study, we could only use two oocytes for IVF because of a limited number of sheep with restored ovarian function after autotransplantation. Thus, more animals should be used in future experiments to obtain more oocytes for the IVF procedure.

In the current study, restoration of follicle function was manifested by production of E2 in autotransplanted ewes. In fact, elevated E2 concentration during FSH-treatment was visible in autotransplanted ewes, indicating the presence of functional developing follicles. Moreover, elevated peripheral FSH concentration in autotransplanted ewes, compared with control values, in the period between ovarian transplantation and FSH treatment is typical for ewes with follicles that are not fully functional [21,23,24,26].

In the current study, histological evaluation demonstrated the presence of primordial, primary, secondary, antral and preovulatory follicles, and well-developed vascularization in both autotransplanted and control ovaries. Previously, we and others have reported a similar morphology in frozen/thawed and fresh ovaries from sheep [24,30]. Moreover, in humans, a similar ovarian morphology was observed in freshly removed ovaries, and in ovaries that had cryoprotectant exposure before freezing or after freezing/thawing [6]. In contrast, Fabbri et al. [16] reported that follicle morphology was affected by the type of cryoprotectant and donor's age. Furthermore, several studies demonstrated altered ovarian morphology after freezing/thawing in humans and sheep [9,27]. Such diverse results are likely due to the different freezing/thawing protocols, and/or transplantation procedures.

The pattern of staining for factor VIII and VEGF (markers of blood vessels) and SMCA (marker of smooth muscle cells) was similar for autotransplanted and control ovaries in our study. This clearly demonstrates that angiogenesis, vascularization, expression of smooth muscle cells in stromal tissue and in blood vessels were fully restored. Arav et al. [30] also demonstrated that cryopreserved and transplanted ovine whole ovaries were expressing factor VIII. Thus, it appears that vascularization and stromal tissue functions, which are critical to support growth and development of ovarian follicles and healthy oocytes, can be restored to normal function after freezing/thawing and autotransplantation.

Additionally, our data demonstrated that there was similar expression of proliferating and apoptotic cells within preovulatory follicles and the CL of both autotransplanted and control ovaries indicating that autotransplanted ovaries have restored their function. These data are similar to those we have previously reported describing the pattern of follicular cell proliferation in normal cycling sheep, and suggest that follicular development and growth of other cells in autotransplanted ovaries can be restored [52]. The expression pattern of apoptotic cells within ovarian follicles in frozen/thawed autotransplanted ovaries in this study was similar to those previously reported for whole frozen/thawed autotransplanted ovaries of sheep [24] and of humans [7]. In contrast, others demonstrated that the apoptotic process was enhanced after freezing/thawing compared with fresh human ovarian cortical tissues [9,18]. In the present study we did not determine the rate of apoptosis immediately before or after freezing/thawing, but it is likely that after autotransplantation, during the restoration of ovarian function, apoptotic cells were removed from ovarian tissues through phagocytosis or other processes. Furthermore, the apoptosis detected in preovulatry follicles and the CL in autotransplanted ovaries is typical of that in normal ovaries [40,42]. Thus, it seems that the balance between proliferation and apoptosis was maintained in autotransplanted ovaries indicating normal physiological functions.

In our previous work on transplantation of whole fresh and frozen ovaries to a heterotopic site we demonstrated technically feasibility and restoration of ovarian function. However, restoration of ovarian function was demonstrated simply by measuring gonadotropins and estrogen and oocyte survival, but not oocyte competence to progress to metaphase stage. Thus, in this longer-term study we have performed more complex characterization of frozen/thawed autotransplanted ovaries than in our previous shorter-term studies [5,14,17,23,24]. In summary, the current study demonstrated that ovarian function was partially restored after heterotopic autotransplantation of frozen/thawed ovaries, since 1) development of preovulatory follicles was stimulated by in vivo FSH treatment, 2) healthy looking oocytes were collected and were matured in vitro, 3) follicles from several stages of folliculogenesis were present, 4) blood vessels expressing specific markers of vascularization were present, indicating active angiogenesis, 5) ovarian cell proliferation was expressed, and 6) apoptosis in ovarian tissues was minimal. Thus, autotransplantation of an intact frozen-thawed ovary is feasible because vascularization and cellular function may be restored. However, since the efficacy of this procedure is low (e.g., in our study a few oocytes were collected, and 25% of autotransplanted ovaries partially restored their function), further improvements are required to enhance the efficiency of autotransplantation of frozen/thawed whole ovaries.

## Competing interests

The author(s) declare that they have no competing interests.

## Authors' contributions

ATGB prepared the manuscript for publication, coordinated and supervised animal care and all laboratory evaluations at the Department of Animal Sciences, NDSU. JB participated in the actual conduct of the experiment, coordinating in the microsurgical procedure, transportation and freezing of the ovaries and reimplantation of the ovaries, pre- and post-surgical care of the animals at Cleveland Clinic, participated in tissue collection and evaluation conducted at NDSU, and participated in preparation of the manuscript. IY conducted the actual surgeries and all the microsurgical procedures before and after cryopreservation of the ovaries. EB assisted in ovarian collection, performed oocyte separation and in vitro fertilization, fixed tissues, performed hormone assays, and helped to draft the manuscript. JJB was responsible for animal care at NDSU, assisted in ovarian collection, performed histological and immunohistological evaluations, and generated images of stained sections. RKS participated in designing of the study, coordinated the experiment at Cleveland Clinic, evaluated results, and participated in writing the manuscript and intellectual content of the study. MS coordinated the designing of all the microsurgical aspects of the procedures, troubleshooting of the challenges during and after microsurgeries along with IY. TF designed of the study, coordinated the experiment at Cleveland Clinic, participated in the actual surgical procedures, was responsible for intellectual content of the study, evaluation of results and writing the manuscript. All authors read and approved the final manuscript.
